# Septic History Limits the Outcome of Tibiotalocalcaneal Arthrodesis

**DOI:** 10.3390/jcm12103422

**Published:** 2023-05-12

**Authors:** Magalie Meinert, Christian Colcuc, Eva Herrmann, Johannes Harbering, Yves Gramlich, Marc Blank, Reinhard Hoffmann, Sebastian Fischer

**Affiliations:** 1 Department for Trauma and Orthopaedic Surgery, Berufsgenossenschaftliche Unfallklinik Frankfurt am Main, 60389 Frankfurt, Germany; 2 Department for Trauma and Orthopaedic Surgery, Evangelical Hospital Bethel Bielefeld, 33611 Bielefeld, Germany; 3 Division of Biostatistics and Mathematical Modelling, Goethe-University, Frankfurt am Main, Theodor-Stern-Kai 7, 60596 Frankfurt am Main, Germany; 4 Department for Septic Bone Surgery, Berufsgenossenschaftliche Unfallklinik Frankfurt am Main, 60389 Frankfurt, Germany; 5 Department of Foot and Ankle Surgery, Berufsgenossenschaftliche Unfallklinik Frankfurt am Main, 60389 Frankfurt, Germany

**Keywords:** ankle arthrodesis, tibiotalocalcaneal arthrodesis, septic history, hindfoot fusion nail, posttraumatic septic osteoarthritis

## Abstract

Joint destruction necessitates tibiotalocalcaneal arthrodesis (TTCA) in cases of clinical deficits that cannot be controlled conservatively, possibly leading to sepsis. We aimed to compare the underlying etiology of posttraumatic joint destruction and the outcomes after TTCA in patients with a septic or aseptic history. Between 2010 and 2022, 216 patients with TTCA were retrospectively enrolled (septic TTCA (S-TTCA) = 129; aseptic TTCA (A-TTCA) = 87). Patient demographics, etiology, Olerud and Molander Ankle Scores (OMASs), Foot Function Index (FFI-D) scores, and Short Form-12 Questionnaire (SF-12) scores were recorded. The mean follow-up period was 6.5 years. Tibial plafond and ankle fractures were the most common causes of sepsis. The mean OMAS was 43.0; the mean FFI-D was 76.7; and the mean SF-12 physical component summary score was 35.5. All the scores differed significantly between the groups (*p* < 0.001). With an average of 11 operations until the arthrodesis was achieved, the S-TTCA patients underwent about three times as many operations as the A-TTCA patients (*p* < 0.001), and 41% of S-TTCA patients remained permanently unable to work (*p* < 0.001). The significantly worse results of S-TTCA compared to A-TTCA show the long and stressful ordeal that patients with a septic history suffer. Further attention must be paid to infection prophylaxis and, if necessary, early infection revision.

## 1. Introduction

Tibiotalocalcaneal arthrodesis (TTCA) is usually the last resort after severe destruction of the ankle joint to relieve the affected patient’s pain and restore stability. However, infections that cannot be controlled after ankle fractures can often only be healed in this way. In this case, freedom from infection and pain relief are preferred to the preservation of ankle function.

Regarding histories of septic TTCA (S-TTCA) and aseptic TTCA (A-TTCA), infections represent S-TTCA due to underlying open fractures or operative fracture treatment [1]. However, elective surgery to address chronic ligament instability, chronic syndesmotic instability, nonunion revisions, lower limb malpositions, and failed total ankle replacements may also result in a septic situation and require a TTCA [2,3]. Primary arthrosis of of all ankle arthroses plays only a minor role, as does hematogenous infection [3,4]. It is well known that TTCA represents an already massive impairment to quality of life and must not be indicated too generously [5,6].

We aimed to determine the level of impairment following TTCA for the treatment of end-stage posttraumatic osteoarthritis of the ankle with a history of sepsis and to compare it with the level of impairment after aseptic TTCA. The selected scores (Olerud and Molander Ankle Score (OMAS), Foot Function Index (FFI-D), and Short Form-12 Questionnaire) were intended to improve the understanding of how to cope with daily life after TTCA and to objectify the influence of the septic history.Our results may assist in the planning and implementation of the surgical procedure with appropriate care and adaption at an early stage to prepare the patient for the expected lengthy treatment.

## 2. Patients and Methods

### 2.1. Population

**Figure 1:**
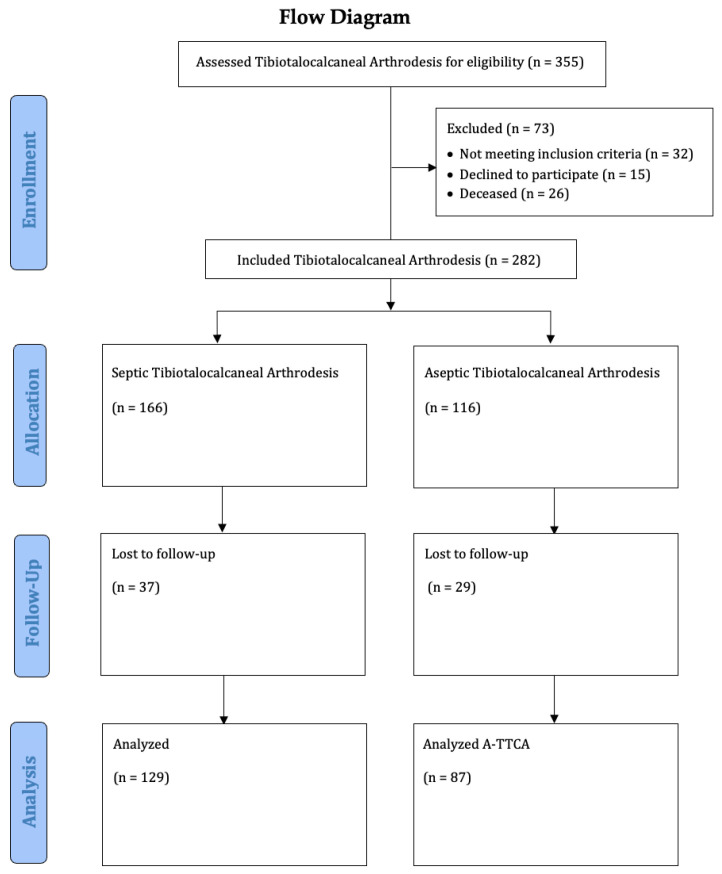
Study flow chart.

Between 2010 and 2022, 216 patients with TTCA due to posttraumatic osteoarthritis (135 males and 81 females; mean age: 64 years (range: 27–93 years)) were retrospectively enrolled in this comparative monocentric study. In total, 129 patients suffered a septic history (S-TTCA) until the fusion of the arthrodesis; 87 had an aseptic history (A-TTCA). Regarding demographics, both groups were equally distributed (Table 1). All patients were seen at our study center (Figure 1). In line with the focus of the study center, approximately one-third of the patients with septic histories were admitted from other hospitals. All arthrodeses were then performed at our study center, involving five surgeons with the same amount of expertise in this type of surgery.

The mean follow-up duration for clinical outcomes was 6.5 years (range: 12–154 months). All procedures were performed in accordance with the 1964 Helsinki Declaration and its later amendments. The ethics committee of the institutional review board approved this study.

### 2.2. Inclusion and Exclusion Criteria

Only patients older than 18 years of age were included. There was no maximum age limit. Written informed consent was required prior to participation. The indication for TTCA was based on underlying painful, end-stage septic or aseptic osteoarthritis of the ankle. Only TTCAs performed at the study center were included.

Destruction of the ankle joint due to malignant neoplasms of bone, such as osteosarcoma, were excluded. Patients who required (partial) amputation of the affected limb as part of the septic history were also excluded.

### 2.3. Surgical Procedure

**Figure 2:**
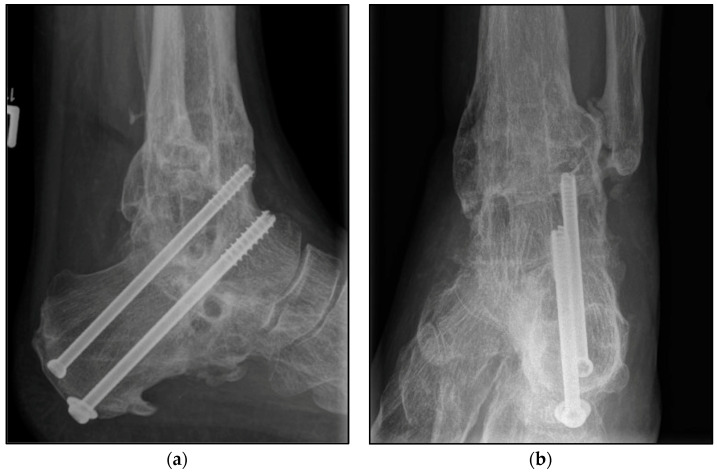
Postoperative radiographic findings of end-stage posttraumatic arthritis of the left ankle with septic history of a 54-year-old male treated with a screw fixation due to nonunion after tibiotalocalcaneal arthrodesis treated with arthrodesis nail. (**a**,**b**) Anteroposterior and lateral view; view, 6 years post operation.

**Figure 3:**
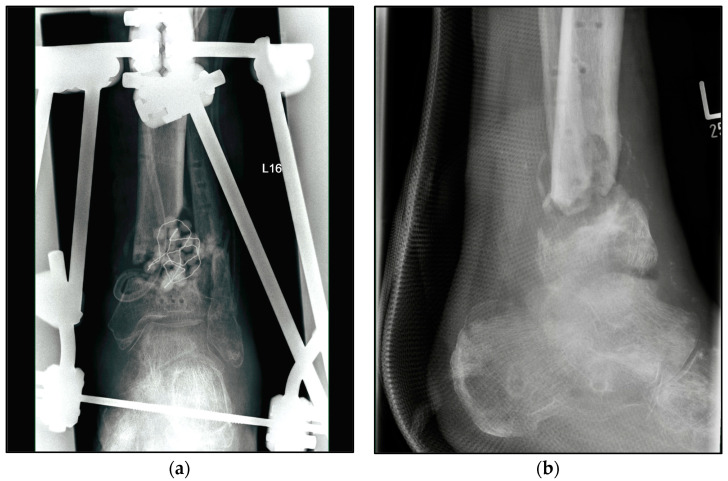
Radiographic findings of the left ankle of a 82-year-old male patient with condition according to open 3° tibial fracture with septic history and treated with external fixator. (**a**) Anteroposterior view; (**b**) lateral view.

**Figure 4:**
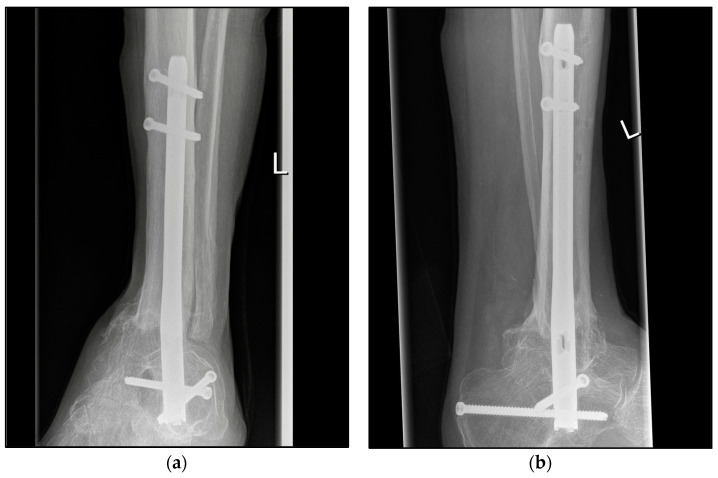
Postoperative radiographic findings of end-stage posttraumatic arthritis of the left ankle with septic history of a 82-year-old male treated with a tibiotalocalcaneal arthrodesis T2™ Ankle Arthrodesis Nail, 200 × 10 mm. (**a**,**b**) Anteroposterior and lateral view; view, 5 years post operation.

The choice of osteosynthetic procedure was based on the experience of the surgeon as well as the intraoperative findings. In the S-TTCA group, approximately 70% of the ankle arthrodesis procedures were performed with a hindfoot fusion nail with 5° of valgus, approximately 20% with an external fixator, and 10% with screws and wires (Figure 2). Procedure changes from nail to fixator or vice versa were often necessary (Figure 3). Depending on the focus of the septic history, all common approaches to the ankle and hindfoot were used, with the lateral approach being the most common at over 60%. For hindfoot nails, the diameter and length of the nail were chosen between 150 and 300 mm according to preoperative planning and intraoperative findings (Figure 4). A shorter nail with a diameter of 12 mm was the most common version.

In the case of a history of infection, in addition to the surgical treatment of the infection, an accompanying antibiotic therapy was regularly carried out. The antibiotics were discontinued 2–4 weeks after the insertion of the nail during the last revision and the receipt of a negative microbiological result. Individual decisions varied depending on the clinical findings.

In the A-TTCA group, almost all arthrodeses were performed with a hindfoot fusion nail with 5° of valgus (predominantly with a T2™ Ankle Arthrodesis Nail, © Stryker, Kalamazoo, MI, USA). The lateral approach was the most frequent at over 80%, followed by approximately 10% of the ventral approach with the use of a fusion plate at the tibiotalar joint in combination with mini-open arthrodesis using screw fixation at the subtalar joint. The interposition of autogenous or autologous cancellous bone grafting was performed in approximately 15% and 25% of the S-TTCA and A-TTCA procedures, respectively.

### 2.4. Rehabilitation Protocol

After the last stage of revision for the treatment of infection, the post-treatment scheme involved wearing an orthotic boot (e.g., VACOped™, OPED GmbH, Valley, Germany) for a total of 12 weeks and ambulation on forearm or armpit crutches for all patients. For the first 6 weeks, patients were required to wear the boot for 24 h per day with merely sole contact; the removal of the boot for personal hygiene and physiotherapy was permitted. After X-ray examination, the boot was worn for an additional 6 weeks with gradual weight bearing; during this time, the boot could be removed at night. At 12 weeks post operation, computed tomography was carried out, and the footwear was orthopedically adapted for everyday use.

### 2.5. Assessment Methods

Demographic data, including age, body mass index (BMI), pre-existing conditions, such as those associated with syndrome-x, and nicotine abuse, were obtained for each patient. Additionally, the underlying etiology of joint destruction, accident mechanism, if applicable, type of fracture and tissue damage according to the Gustilo grade I–III classification, and the outcome using the Olerud and Molander Ankle Score (OMAS), Foot Function Index in its validated German version (FFI-D), and Short Form-12 Questionnaire (SF-12) were recorded. The type and number of revisions were also recorded as part of the follow-up (Table 2).

### 2.6. Statistical Analysis

The primary aim was to compare significant differences in the outcomes of S-TTCA and A-TTCA using a representative number of cases which illustrated the power of the included data with a mean follow-up time of 6.5 years. Due to the retrospective design, there was no case number calculation. So far, monocentric studies with comparable questions have tended to have smaller population groups [7,8,9]. All the statistical analyses were performed using SPSS, v. 23, software (IBM Dtl. GmbH, Ehningen, Germany). Furthermore, for descriptive and explorative statistical analyses for the queried scores, including within-group means, medians, minima and maxima, and standard deviations, Student’s *t*-test and an ANOVA were used. The power of the study was 0.8, and the significance level was set to *p* < 0.05 with a 95% confidence interval.

## 3. Results

After an average postoperative follow-up of 6.5 years (range: 12–154 months) the following factors were identified as the causes of the terminal posttraumatic arthritis of the ankle. At 36.4% and 33.3%, tibial plafond fractures and ankle fractures were the most common injuries with septic complications, respectively; in approximately 25% of the cases, the underlying fracture was an open 3° fracture. The proportion of the other causes of end-stage posttraumatic osteoarthritis was 23% in the S-TTCA group, predominantly combined with soft tissue damage; in the A-TTCA group, this proportion was 12% and was due to chronic syndesmotic instability in half of the cases. Multiple answers were possible in both groups.

The mean OMAS was 43.0 (S-TTCA: 39.4, A-TTCA: 48.4); the mean FFI-D was 76.7 (S-TTCA: 81.6, A-TTCA: 69.2); and the mean SF-12 physical component summary score was 35.5 (S-TTCA: 34.1, A-TTCA: 37.7). All the scores differed significantly between the groups (*p* < 0.001). The SF-12 mental component summary, on the other hand, did not show any significant differences (mean: 50.1, S-TTCA: 49.9, A-TTCA: 50.4, *p* = 0.783).

With an average of 11 operations until union of the arthrodesis was achieved, the S-TTCA patients underwent approximately 3 times as many operations as the A-TTCA patients (*p* < 0.001). Approximately 41% of S-TTCA cases remained permanently unable to work, compared to approximately 18% percent of the A-TTCA group (*p* < 0.001) (Table 3).

### Complications

Since all patients in the S-TTCA group underwent multiple revisions, including soft tissue debridement as well as nonunion revisions due to persistent infection, these procedures were not listed separately as complications. The overall revision rate of the A-TTCA group was approximately 16%. Revisions had to be performed due to nonunion in eight cases; five revisions were for implant irrigation. Minor complications such as delayed wound healing, swelling, discomfort, and cramps were seen in both treatment groups. Cases not requiring revision were not considered a relevant complication in the present study (Table 3). Another serious difference was found in the time from initial trauma to arthrodesis. This period was 98 months on average in the S-TTCA group and 246 months in the A-TTCA group.

## 4. Discussion

The data obtained from the validated scores confirm the clinical impression of the authors that the outcome of TTCA with a septic history differs significantly from patients without a history of infection, even after clinically and radiologically confirmed healing of the arthrodesis. Although the patients from the S-TTCA group tended to have more metabolic-syndrome-associated previous diseases, this difference was not significant. All other demographic data, such as age, gender, BMI, and smoking status, were equally distributed.

The proportion of metabolic-syndrome-associated pre-existing conditions was significantly higher than in the normal population, as was the average BMI. It is understandable that patients with a history of infections have an increased risk profile due to their lifestyle [10]. However, this cannot be confirmed based on the available data. The well-known correlation between increased BMI and frequency of ankle injuries was also confirmed [11,12]. The accompanying increased rate of metabolic-syndrome-associated diseases could also be seen in this context.

There were an equal number of men in each group. Men have higher rates than women for all crash types and crash-related injuries, not only for the lower limb [13]. Naturally, this is due to the comparatively higher proportion of male involvement in both traffic accidents and falls from height at construction sites, as can often be inferred from the medical history.

In accordance with the focus of the study center, we found accident- and infection-related joint damage to be the cause of terminal posttraumatic arthrosis of the ankle joint. At approximately 36%, pilon fractures were the leading etiology of ankle joint arthrosis, immediately followed by fractures of the ankle joint and the talus itself. At 24.8%, the proportion of open fractures was approximately four times higher in the S-TTCA group than in the A-TTCA; Gustilo grade III fractures were approximately three times as high (S-TTCA: 13.95%, A-TTCA: 4.59%). The comparative literature confirms this proportion of open fractures in the context of ankle fractures to be approximately 13%, depending on the mechanism and complexity following motor vehicle or motorbike collisions and falls from height [14]. Comparative breakdowns by Gustilo grade I–III injury, as in the present work, are generally difficult to find. However, in the case of smooth transitions between the grades, the data should generally be viewed critically, although there is agreement that all open fractures increase the risk of deep wound infection many times over, with a significantly higher number of reoperations, flap reconstructions, and patients suffering from chronic pain when compared to other grades [14,15,16,17].

A remarkable result of our study lies in the period from the first named accident event or ankle osteoarthritis due to rarer causes such as axial malalignment or chronic instability until arthrodesis of the ankle joint or full healing. This period was merely 98 months on average in the S-TTCA group and 246 months in the A-TTCA group. An obvious explanation is that the present infection requires immediate surgical revision but also leads to additional aggressive joint destruction. Studies with comparable questions indicate the period from the designation of septic arthritis to arthrodesis as 30–80 months [18]. For A-TTCA, a period of 8–10 years has been reported [19]. The comparatively longer period, as indicated in the present study, is explained by the designation of the initial trauma and not by the designation of the first evidence of deep infection. Accordingly, the period from the first trauma to the final performance of A-TTCA is significantly longer than is usually stated in comparative studies [20].

The attempt to assess the choice of osteosynthesis procedure led to the following conclusion. The data analysis did not reveal any difference between the individual osteosynthetic procedures, such as nails, screws, and external fixators, which were therefore not presented separately. Again, multiple changes were reported regarding the number of previous operations, from fusion nails to external fixators and vice versa, especially in the S-TTCA group. Therefore, an evaluation of which procedure is superior cannot be made. Again, the data agree with those of the comparative literature and confirm the fusion nail as the method of choice for TTCA in the absence of infection. Furthermore, an external fixator such as the Ilizarov frame is a proven alternative, with comparable results in the presence or after the revision of a deep infection [10,21,22,23,24].

The clinical outcome of both the separately presented S-TTCA and the aseptic TTCA, with an average of 33.5 points in the physical component summary (PCS) of the SF-12 Questionnaire and 50.1 points for the mental component summary of the SF-12 Questionnaire, is largely in line with the results of studies with comparable questions [25]. Studies also indicate values above 50 for the PCS of the SF-12 [26]. An obvious explanation is the lack of patients with a history of sepsis. Similarly, studies have evaluated comparatively worse outcomes measured by the OMAS. Fuchs et al. gave a score of 59 points in their 20-year review [27]. In addition to the few representative cases, the proportion of the septic population in that study was just 4/18 (22.2%) and not 129/216 patients (59.7%), as in our present study. There was also no comparison of the two groups. Jonas et al. and Georgiannos et al. present data that appears to be more realistic. Values above 50 points for a purely aseptic population support the original and confirmed assumption of the present study that S-TTCA and A-TTCA differ significantly in outcome [28,29].

In addition to the strength of a large population in this monocentric study, this study had some limitations. First, this was a study with a retrospective design, and the clinical scores and the extent of septic and posttraumatic damage to the tibiotalar and subtalar joints were not collected preoperatively. Based on the retrospectively collected data, it can be determined which cases with septic arthritis would have healed even without arthrodesis or in which cases, viewed retrospectively, the decision was made too early or should have been made earlier. Regardless, all patients equally presented with posttraumatic terminal osteoarthritis of the ankle. Second, regarding the group with a history of sepsis, the complications in the context of the necessary TTCA cannot be reliably distinguished from the multiple revisions for infection treatment and thus provide a direct comparison.

## 5. Conclusions

The available data, with the significantly worse results of S-TTCA compared to A-TTCA in clinical scores and prolonged downtime or permanent incapacity, show the long and stressful ordeal that patients with a septic history suffer. For that reason, more attention should be paid to infection prophylaxis and, if necessary, early infection revision, especially in the context of tibial plafond fractures and even more so in the case of an open fracture.

## Figures and Tables

**Table 1:** Patient characteristics.

Characteristic		Septic TTCA (n = 129)	Aseptic TTCA (n = 87)	All (n = 216)	*p*
Follow-up (months)	Mean	85.10	68.87	78.61	0.003
	SEM	3.36	4.42	2.73	
	Minimum	18.00	12.00	12.00	
	Maximum	154.00	151.00	154.00	
Age, years	Mean	63.59	64.68	64.02	0.528
	SEM	1.02	1.47	0.85	
	Minimum	27.00	30.00	27.00	
	Maximum	89.00	93.00	93.00	
BMI, kg/m^2^	Mean	30.34	29.95	30.18	0.660
	SEM	0.56	0.68	0.43	
	Minimum	16.40	18.80	16.40	
	Maximum	58.30	49.60	58.30	
Sex, n (%)	Male	84 (65.12)	51 (58.62)	135 (62.50)	0.336
	Female	45 (34.88)	36 (41.38)	81 (37.50)	
Affected side, n (%)	Left	67 (51.94)	44 (50.58)	111 (51.39)	0.845
	Right	62 (48.06)	43 (49.42)	105 (48.61)	
Smoker, n (%)	Yes	34 (26.36)	17 (19.54)	51 (23.61)	0.266
	No	92 (71.32)	67 (77.01)	159 (73.61)	
	n.a.	3 (2.33)	3 (3.45)	6 (2.78)	
Pre-existing conditions (multiple answers), n (%)	Metabolic-syndrome-associated	52 (41.27)	30 (35.71)	82 (37.96)	0.063
	Rheumatism	7 (5.43)	3 (3.45)	10 (4.63)	
	Others	25 (19.38)	19 (21.84)	44 (20.37)	
	None	30 (23.26)	15 (17.24)	45 (20.83)	

BMI, body mass index; SEM, standard error of the mean; TTCA, tibiotalocalcaneal arthrodesis.

**Table 2:** Etiology of the underlying end-stage posttraumatic osteoarthrosis of the ankle joint.

Predisposing Factors, Multiple Answers		Septic TTCA (n = 129)	Aseptic TTCA (n = 87)	All (n = 216)	*p*
Mechanism, n (%)	Fall from height	43 (33.33)	26 (29.89)	69 (31.94)	0.697
	Distortion	29 (22.48)	14 (16.09)	43 (19.91)	
	Traffic accident	30 (23.26)	21 (24.14)	51 (23.61)	
	Others	26 (20.63)	26 (29.89)	52 (24.07)	
Ankle fracture, n (%)	Yes	43 (33.33)	25 (28.74)	68 (31.48)	0.478
	No	86 (66.67)	62 (71.27)	148 (68.52)	
Talar fracture, n (%)	Yes	6 (4.65)	18 (20.70)	24 (11.11)	0.001
	No	123 (95.35)	69 (79.30)	192 (88.89)	
Tibial plafond fractures, n (%)	Yes	47 (36.43)	5 (5.75)	52 (24.08)	0.001
	No	82 (63.57)	82 (94.25)	164 (75.92)	
Open fracture, n (%)	Yes	32 (24.81)	7 (8.05)	39 (18.06)	<0.001
	No	97 (75.19)	80 (91.95)	177 (81.94)	
	Gustilo Grade I	2 (1.55)	0 (0.00)	2 (0.93)	
	Gustilo Grade II	12 (9.30)	3 (3.45)	15 (6.94)	
	Gustilo Grade III	18 (13.95)	4 (4.59)	22 (10.19)	
Delayed union tibial, n (%)	Yes	58 (44.96)	17 (19.54)	75 (34.72)	<0.001
	No	71 (55.04)	70 (80.46)	141 (65.28)	
Chronic ankle instability, n (%)	Yes	8 (6.20)	7 (8.05)	15 (6.94)	0.603
	No	121 (93.80)	80 (91.95)	201 (93.06)	
Chronic syndesmotic instability, n (%)	Yes	4 (3.10)	6 (6.90)	10 (4.63)	0.120
	No	125 (96.90)	81 (93.10)	206 (95.37)	
Failed total ankle replacement, n (%)	Yes	17 (13.18)	12 (13.79)	29 (13.43)	0.897
	No	112 (86.82)	75 (86.21)	187 (86.57)	
Deformities of the lower limb, n (%)	Valgus deformity	1 (0.78)	9 (10.35)	10 (4.63)	<0.001
	Varus deformity	6 (4.65)	23 (26.44)	29 (13.43)	<0.001
	No	122 (94.57)	55 (63.21)	177 (81.94)	<0.001
Previous neurological disease, n (%)	Yes	4 (3.10)	6 (6.90)	10 (4.63)	0.195
	No	125 (96.90)	81 (93.10)	206 (95.37)	
Primary arthrosis, n (%)	Yes	3 (2.33)	6 (6.90)	9 (4.17)	0.100
	No	126 (97.67)	81 (93.10)	207 (95.83)	
Others, n (%)	Yes	23 (17.83)	14 (16.01)	37 (17.13)	0.735
	No	106 (82.17)	73 (83.99)	179 (82.87)	

TTCA, tibiotalocalcaneal arthrodesis.

**Table 3:** Clinical outcome with subgroups.

Measurements		Septic TTCA (n = 129)	Aseptic TTCA (n = 87)	All (n = 216)	*p*
Olerud and Molander	Mean	39.40	48.39	43.00	0.008
	SEM	2.08	2.72	1.68	
	Minimum	0.00	0.00	0.00	
	Maximum	100.00	85.00	100.00	
FFI-D	Mean	81.62	69.23	76.64	0.002
	SEM	2.49	3.11	1.98	
	Minimum	19.50	15.00	15.00	
	Maximum	135.00	123.00	135.00	
SF-12 (physical component summary)	Mean	34.09	37.66	35.52	0.019
	SEM	0.96	1.14	35.52	
	Minimum	11.73	14.47	0.75	
	Maximum	55.26	56.63	11.73	
SF-12 (mental component summary)	Mean	49.91	50.36	50.09	0.783
	SEM	1.11	1.19	0.814	
	Minimum	17.10	22.84	17.10	
	Maximum	68.89	65.23	68.89	
Number of operations underwent until union, including fracture treatment, n	Mean	11.23	3.83	8.29	<0.001
	Minimum	1	1	1	
	Maximum	30	25	30	
Complication, revision surgery needed until union (multiple answers), n (%) *	Yes	-	14 (16.09)		
	No	-	73 (83.91)		
	Nonunion	-	8 (9.19)		
	Implant irritation	-	5 (5.74)		
	TTS	-	1 (1.15)		
Footwear (multiple answers), n (%)	Orthotic insoles only	23 (17.83)	18 (20.69)	41 (18.98)	0.012
	Shoe adaption	72 (55.81)	34 (39.08)	106 (49.07)	<0.001
	Other	2 (1.59)	1 (1.19)	3 (1.39)	0.281
	Nothing special	29 (22.48)	31 (35.63)	60 (27.78)	<0.001
Return to learned profession, n (%)	Yes	18 (13.95)	16 (18.39)	34 (15.74)	0.802
Permanently unable to work, n (%)	Yes	52 (41.27)	15 (17.86)	67 (31.02)	<0.001
Retraining, part time, and pension, n (%)	Yes	56 (44.44)	53 (63.09)	109 (50.46)	0.802

SEM, standard error of the mean; SF-12, 12-Item Short Form Health Survey; TTCA, tibiotalocalcaneal arthrodesis; TTS, tarsal tunnel syndrome. * All patients in the septic TTCA group had revisions due to persistent infections with failure of soft tissue healing and pseudarthrosis. Therefore, these procedures were not considered complications.

## Data Availability

All data intended for publication are included in the manuscript.
